# Recent advances in anterior pituitary hormones and metabolic-associated fatty liver disease

**DOI:** 10.3389/fendo.2025.1600559

**Published:** 2025-07-04

**Authors:** Changqing Liu, Yuanyuan Long, Junjun Liu, Yongfeng Song

**Affiliations:** ^1^ Endocrine and Metabolic Diseases Hospital of Shandong First Medical University, Shandong First Medical University and Shandong Academy of Medical Sciences, Jinan, Shandong, China; ^2^ Department of Endocrinology, Jinan Central Hospital Affiliated to Shandong First Medical University, Jinan, Shandong, China; ^3^ Shandong Clinical Research Center of Diabetes and Metabolic Diseases, Jinan, Shandong, China; ^4^ Shandong Institute of Endocrine and Metabolic Diseases, Shandong First Medical University, Jinan, Shandong, China; ^5^ Shandong Engineering Laboratory of Prevention and Control for Endocrine and Metabolic Diseases, Jinan, Shandong, China

**Keywords:** MAFLD, anterior pituitary hormone, lipid metabolism, metabolism dysfunction, metabolic mechanisms signalling

## Abstract

The classical theory of the pituitary–target gland axis suggests that the hormones secreted by the pituitary gland only regulate the synthesis and secretion of target gland hormones, while the target gland hormones act on the tissues of the body to achieve biological functions. However, recent studies have shown that anterior pituitary hormone receptors are also expressed on the surface of hepatocytes. This suggests that anterior pituitary hormones may act directly on hepatocytes to exert regulatory effects independent of target hormones. The review systematically summarizes the mechanisms and effects of thyroid-stimulating hormone (TSH), follicle-stimulating hormone (FSH), luteinizing hormone (LH), prolactin (PRL), adrenocorticotropic hormone (ACTH), growth hormone (GH), and melanocyte-stimulating hormones (MSH) on liver metabolism and their roles in the pathogenesis of Metabolic-Associated Fatty Liver Disease (MAFLD). It is hoped that this will provide new insights into the prevention and treatment of MAFLD.

## Introduction

As the central regulator of the body’s metabolism, the pituitary plays a crucial role in growth, development, immune function, energy metabolism and reproduction, among other functions. Each anterior pituitary hormone binds to its receptor in its classical target gland, for example the thyroid binds to TSH, to regulate physiological pathways. However, in recent decades, several reports have shown that anterior pituitary hormone receptors not only expressed in the target gland, but also expressed in the non-target gland, such as in the liver. Thus, each hormone exerts an unconventional biological effect in the liver. This review summarizes the functions of anterior pituitary hormones in the liver and their role in the pathogenesis of MAFLD. These findings extend our knowledge of the targets and functions of anterior pituitary hormones and provide new insights into the biological function of anterior pituitary hormones in the onset and development of MAFLD.

## Overview of the MAFLD

MAFLD formerly known as Non-Alcoholic Fatty Liver Disease (NAFLD), has been repeatedly renamed by international medical organizations to reflect its involvement in various pathological conditions and has recently been redefined as Metabolic-Associated Steatotic Liver Disease (MASLD) (1–3). For the purposes of this article, we will refer to MAFLD. MAFLDs are among the most common liver diseases worldwide and are predicted to be the leading cause for end-stage liver disease in the coming decades (4). The prevalence of MAFLD in the global population is maybe as high as 25% (5), and the estimated combined prevalence of non-alcoholic steatohepatitis (NASH) in histologically confirmed MAFLD patients is 59% (6, 7). Importantly, there is strong clinical evidence that MAFLD is also associated with an increased risk of other diseases outside the liver, including cardiovascular disease and extra-hepatic malignancies (8), ultimately leading to death. MAFLD and its associated complications pose a significant burden on socioeconomic development and public health in China (9).

**Figure 1:**
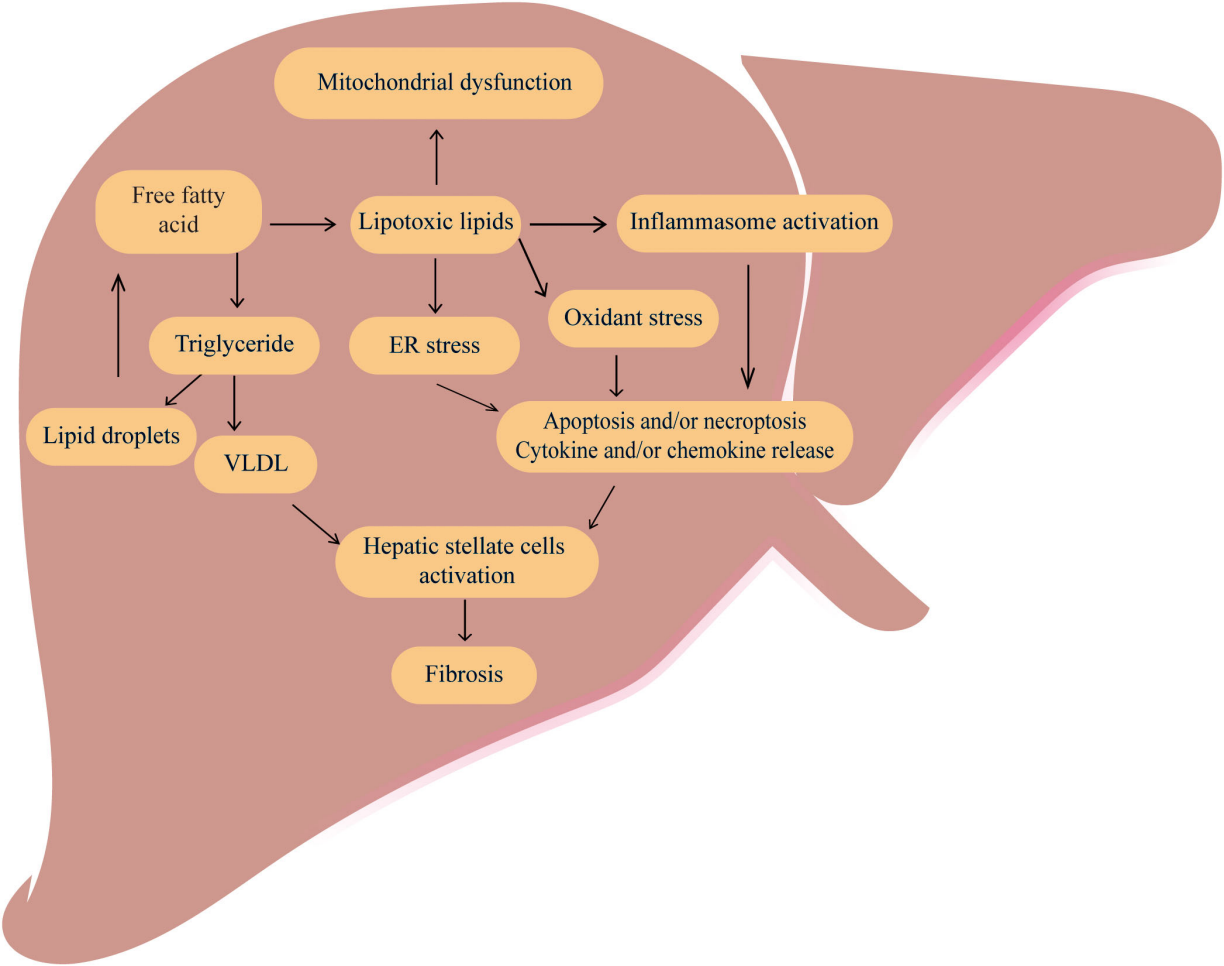
The classic pathogenesis of MAFLD MAFLD is caused by dysregulation of several metabolic processes especially an increased accumulation of free fatty acid and total triglycerides (TG) in the liver. Excessive free fatty acids can cause lipotoxic, ultimately leading to mitochondrial dysfunctions, inflammation, oxidative stress, and endoplasmic reticulum stress, promoting cell apoptosis, necrosis and cytokine release. The VLDL and cytokines can activate hepatic stellate cells and promote the development of liver fibrosis.

MAFLD is a chronic liver disease that is marked by intracytoplasmic lipid accumulation in hepatocytes, and hepatic inflammation and fibrosis (10, 11). And the occurrence of MAFLD is involved in multiple molecular pathways, including hepatic steatosis, insulin resistance, inflammatory cytokines and oxidative stress, apoptosis pathways and adipokines (12). Besides, MAFLD is closely linked to abnormalities in several metabolic processes (13), including glucose metabolism (14), lipid metabolism (15, 16)(**Figure 1**). Free fatty acid (FFA)-mediated-mediated lipotoxicity, cholesterol-induced toxicity and subsequent elevation of reactive oxygen species (ROS) in hepatocytes can promote the development of MALFD (17, 18).

In addition to the above factors, endocrine disruption is an important contributor to hepatic metabolic abnormalities. With advancing age, the function of endocrine glands such as the thyroid and gonads gradually weakens. This leads to a progressive decline in thyroid and sex hormone levels, resulting in significant changes to lipid synthesis, metabolism and degradation processes and ultimately, lipid metabolism disorders. Furthermore, recent research has shown that anterior pituitary hormones can directly influence hepatic lipid metabolism, as well as the processes of inflammation and fibrosis in the liver, independently of the synthesis and secretion of target gland hormones. Therefore, this article will systematically summarize the onset and progression of MAFLD mediated by the imbalance of anterior pituitary hormones through multiple mechanisms, focusing on the following points.

## General description of the anterior pituitary hormones

**Figure 2:**
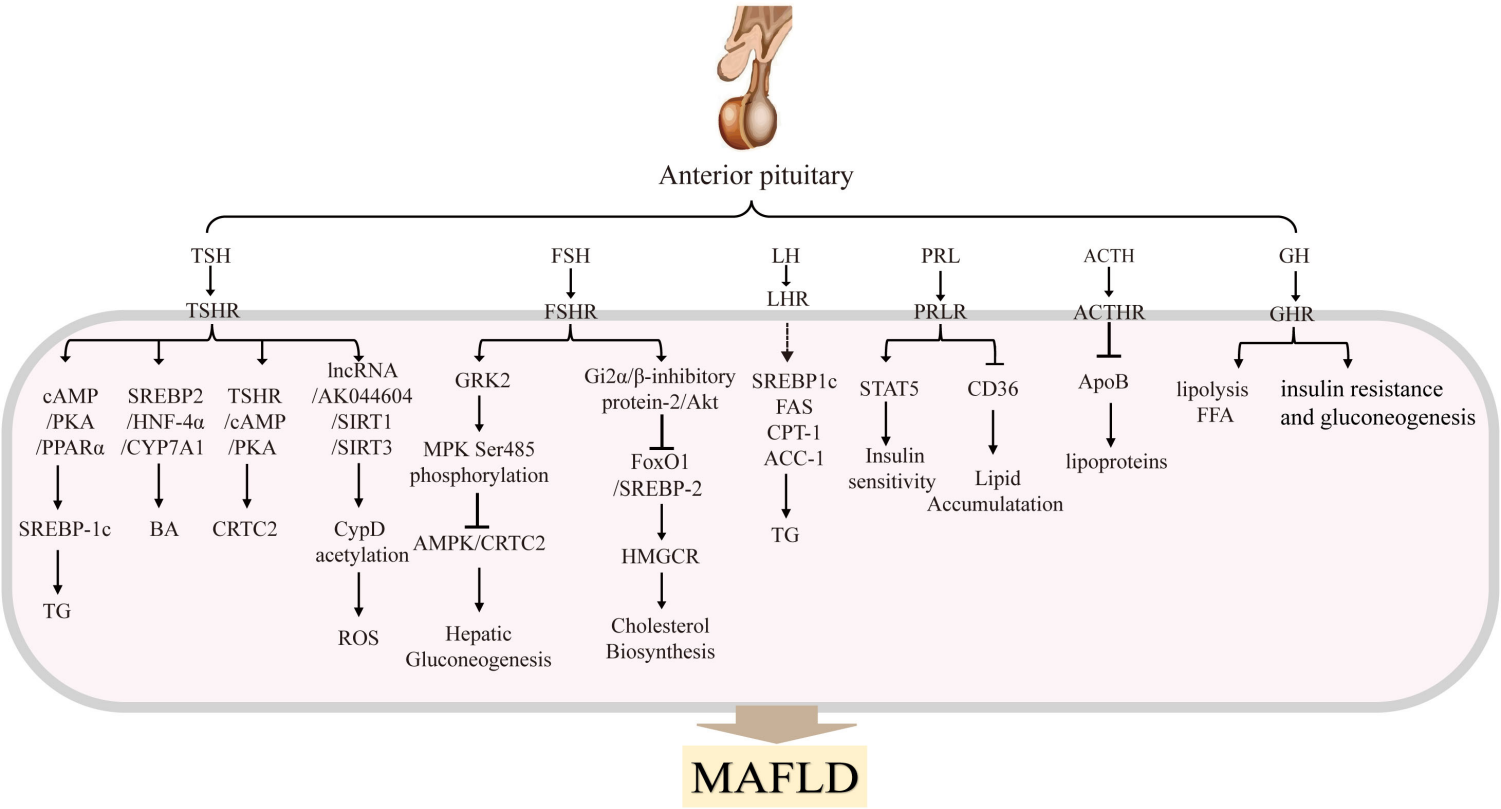
Anterior pituitary hormones and their main effects in MAFLDThe anterior pituitary gland secretes several hormones, including TSH, FSH, LH, PRL, ACTH, GH and MSH. These hormones are transported to the liver, where they bind to their respective receptors and activate signaling pathways that regulate hepatic glucolipid metabolism. These processes influence the progression of MAFLD.

The pituitary plays a central role as a central regulator of metabolism in the body. The pituitary gland, included anterior pituitary and posterior pituitary gland (19). The anterior pituitary secretes TSH, FSH, LH, PRL, ACTH, GH, MSH. By contrast, the posterior pituitary gland secretes two nonapeptides, oxytocin (regulates parturition and lactation) and arginine vasopressin (AVP; controls water reabsorption in the kidney). Here we focus on anterior pituitary hormones. There are three main axis including hypothalamic-pituitary-gonadal (HPG) (20), hypothalamic-pituitary-thyroid (HPT) (21), and hypothalamic-pituitary-adrenal (HPA) axes (22). They influence different organs and tissues in the body by secreting variety of cytokines, growth factors and receptors. Specifically, the HPG axis primarily regulates processes such as sexual development, spermatogenesis and oocyte maturation. It contributes to the establishment and maintenance of the secondary sex characteristics, mainly through the release of LH and FSH. The HPT axis primarily regulates the levels of thyroid hormones and thus participates in regulating the overall metabolism, mainly through the secretion of TSH (23). The HPA axis is primarily involved in controlling stress and several physiological activities including digestion, mood and emotion, which include the main hormone is ACTH. As shown in **Figure 2**, in addition to the hormones secreted by the above three pituitary axes, PRL, GH and MSH are also secreted by the anterior pituitary (22).

## The major effects of pituitary hormone in MAFLD

### TSH

TSH is formed by α and β subunits, and it exerts its important functions by targeting the TSH receptor (TSHR), thereby regulating thyroid hormone levels. TSH plays an important role in metabolism, development, and growth (24). TSHRs are primarily found in the thyroid gland. However, researchers have also discovered their presence in various non-thyroid tissues, such as the liver, adipose tissue, myocardium, bone, thymus, and natural killer (NK) cells (25–31).

The different TSH levels reflect different thyroid status, and it is important to note that both subclinical hypothyroidism (SCH)and overt hypothyroidism are associated with NASH and advanced fibrosis (32–34). In biopsy-proven MAFLD patients with normal thyroid function, there is a clear relationship between high normal TSH levels and NASH, which may be related to the PNPLA3 G risk allele (35). Another study found a positive relationship between TSH levels and NASH in MAFLD patients with thyroid function normal (36). Meanwhile liver biopsies have revealed a significant correlation between TSH levels (32), including macrovesicular steatosis, inflammation, or hepatocyte balloon degeneration and steatosis. These clinical studies have shown that TSH levels are positively correlated with ALT and AST, with a significant reduction in risk when TSH is < 2.5 mIU/mL, but TSH concentrations >2.5mIU/L are associated with an increased risk of lipid and carbohydrate metabolism disorders (32). This has led to widespread discussion in clinical research as to whether a sufficiently low TSH level can prevent the development of MAFLD. As a result, TSH maybe as an independent risk factor for MAFLD, and the risk of MAFLD increases significantly with elevated TSH levels.

TSH in hepatocytes can regulate hepatic lipid and cholesterol metabolism, as shown in **Figure 2**. adenosine monophosphate-activated protein kinase (AMPK) is a key regulator of lipid and glucose metabolism, and reduced AMPK activity increases the expression of genes related to lipid synthesis and cholesterol biosynthesis. Our research has shown that TSH-TSHRs activate the cAMP/PKA/CREB signaling system in hepatocytes and directly promote cholesterol synthesis (37, 38). Furthermore, our research has demonstrated that abnormally elevated TSH, via TSHR, triggers SREBP-1c via the cAMP/PKA/PPARα pathway, thereby increasing hepatic lipid accumulation and ultimately leading to the onset of MAFLD (39). Cell experiments have also shown that TSH inhibits hepatic bile acid synthesis via the SREBP2/HNF-4α/CYP7A1 pathway (40). TSH can also attenuate hepatic fatty acid oxidation by decreasing the mitochondrial distribution of miR-449a/449b-5p/5194. This inhibits fatty acid (FA) cleavage and enhances triglyceride storage in hepatocytes (41). This is the most well-known pathogenic mechanism of TSH-mediated MAFLD.

In addition to affecting hepatic lipid and cholesterol metabolism, TSH may also contribute to the onset and progression of MAFLD by influencing glucose metabolism pathways. Previous reports have suggested that TSH acts directly on gluconeogenesis, with TSH directly regulating hepatic gluconeogenesis in HepG2 cells and Tshr-KO mice (42). More recent research has demonstrated that TSH can increase the expression of the CRTC2 gene and thereby upregulate hepatic gluconeogenic genes via the TSHR/cAMP/PKA pathway (43).

TSH may also mediate MAFLD via oxidative stress and inflammation. Animal studies using TSHR knockout and liver-specific knockout mice, revealed that TSH stimulates CypD acetylation in the liver via the lncRNA-AK044604/SIRT1/SIRT3 pathway (44). This study demonstrated the significant role of TSH in hepatic mitochondrial oxidative stress, leading to the generation of ROS and mediating the onset of MAFLD. In addition, TSH increases the secretion of exosomes by hepatocytes and alters their protein profile. Many of these proteins are required for metabolism, signal transduction, cell apoptosis and inflammation (45). In the obese mice, fed a high-fat diet (HFD), had significantly higher serum TSH levels. Elevated TSH levels lead to increased secretion of SPP1 in M1 macrophages and exacerbate lipid accumulation in hepatocytes (46).

Taken together, these findings suggest that TSH plays an intrinsic role in regulating liver lipid and cholesterol homeostasis. In addition to the liver, TSH may contribute to MAFLD through other organs and tissues. For instance, recent studies have shown that TSH can directly suppress the ATGL gene expression in mature mouse adipocytes via activating cAMP/PKA pathway, thereby inhibiting the basal breakdown of triglycerides (47). On the other hand, through the AMPK/PRDM16/PGC1α pathway, TSHR knockout induced a reduction in adiposity, increased energy expenditure, and promoted the development of beige adipocytes in mouse adipose tissue.

Therefore, TSH is a significant independent risk factor for MAFLD, with the risk increasing significantly with elevated TSH levels. TSH may mediate the onset and progression of MAFLD through various mechanisms, so controlling TSH levels should be taken into consideration as a possible future therapeutic strategy for MAFLD.

### FSH

Both FSH and LH are gonadotropins, which play a role in promoting follicular development and maturation and stimulating estrogen secretion (48). FSH binds to the FSH receptor (FSHR) to exert its regulatory functions (48). FSH receptors (FSHR) are found not only in the ovaries and testes (49), but also in monocytes and osteoclasts (50), adipocytes (51), hepatocytes (52), blood vessels and maybe other tissues (53). In addition, FSH has been found to be associated with insulin sensitivity, bone metabolism, adipogenesis, inflammation, thermogenesis, osteogenesis and ovarian cancer (54). Most directly, liver and adipose can be directly regulated by FSH (55).

Numerous articles have examined the association between FSH and MAFLD in clinical investigations, producing conflicting results. For example, Wang et al. found that FSH levels were negatively associated with the prevalence of MAFLD in Chinese women over 55 years of age, independent of traditional metabolic risk factors such as BMI, glucose and lipids (56). Ge et al. also demonstrated that, in postmenopausal women with an average age of 60.22 ± 6.49 and type 2 diabetes mellitus, FSH was negatively and independently associated with MAFLD (57). A retrospective observational study of a Chinese elderly population, including both men and women aged 60–70 years and over 70 years, also showed a negative correlation between FSH and MAFLD (58). The above results showed that there is a negative correlation between FSH and MAFLD. However, another study of elderly Chinese men aged 80–98 showed a positive correlation between FSH and MAFLD (59), the study showed that a low FSH level may decrease the risk of MAFLD. Furthermore, another study showed that FSH acts on the pituitary corticotropes to inhibit corticosterone production and ultimately prevent hepatic steatosis, and that FSH administration is sufficient to improve metabolic disorders including hepatic steatosis in female mice (55). The reasons for the variation in research outcomes concerning the relationship between FSH and MAFLD are unclear. However, age may have some impact on these differences. Therefore, more well-designed clinical research studies are necessary to confirm the findings.

FSH exerts a significant influence on the body’s lipid metabolism (**Figure 2**). For example, Song et al. (2016) found that postmenopausal women with higher serum FSH (78.3 IU/L at baseline) had higher serum total cholesterol and LDL-C levels, and that there was a significant improvement in lipid levels after hormone replacement therapy or blocking FSH signaling by anti-FSHβ antibody or ablating the FSH receptor (FSHR) gene (52, 60). The main mechanism may be that FSH blocks FoxO1 binding to the sterol regulatory element-binding protein-2 (SREBP-2) promoter through activation of the Gi2α/β inhibitor protein-2/Akt pathway. This effect leads to an increase in SREBP-2, which drives *de novo* transcription of 3-hydroxy-3-methylglutaryl coenzyme A reductase (HMGCR) and increases cholesterol accumulation (60). These researches suggest that blocking FSH signaling may be a new strategy for treating menopausal hypercholesterolemia, particularly in peri-menopausal women characterized by elevated FSH levels.

FSH also plays a role in MAFLD by affecting hepatic gluconeogenesis in hepatocytes (**Figure 2**). Our researches provided evidence that FSH plays a direct role in causing gluconeogenesis, where FSH through FSHR targets GRK2 in the liver, increases AMPK Ser485 phosphorylation to inhibit AMPK activation, and then increases the transcription of hepatic PEPCK and G6pase through CRTC2, thereby enhancing hepatic gluconeogenesis independent of estrogen (61, 62). Beside we have identified a role for FSH in fasting serum glucose levels using FSH receptor knockout mice (61) and the association between FSH levels and insulin resistance has been confirmed in postmenopausal women (63). The relationship between IR and FSH levels may be mediated by regulation of glucocorticoid receptor (GCR) expression (64).

All this provides new insights into the role of FSH in lipid and glucose metabolism and demonstrates the direct involvement of FSH in MAFLD.

### LH

LH stimulates the secretion of the testes and ovaries, whose the classical role is to regulate ovarian and testicular function (65–69)). The receptor of LH (LHR) was expressed in the pineal gland, pituitary, hypothalamus, gonad, kidney, brain, lymphatic tissue and lymphocytes (70, 71).

Although it has been shown that LH plays an important role in the reproductive, urinary and nervous system (72) (73), such as in the porcine oviduct, urinary tract, and hippocampus, there is no evidence of direct relationship between LH and liver or lipid metabolism. However, it is noteworthy that LH beta mRNA expression was elevated in fasted wild-type mice, but not in mice deficient in PPARalpha (74). This research suggests that LH may play a role in lipid metabolism, as PPAR-α is an important transcription factor involved in lipid metabolism.

Notably LH levels are significantly elevated in female populations with PCOS (polycystic ovary syndrome). And abnormal levels of phosphatidylcholine, FFAs and polyunsaturated fatty acid (PUFA) metabolites were found in patients with PCOS (75). Around 70-80% of patients with PCOS are obese. In addition, the expression of genes involved in lipid metabolism, such as FAS, SREBP1c, ACC-1 and CPT-1, is increased in patients with PCOS (76). From the above evidence we can infer that LH may be involved in lipid metabolism. However, there are no direct studies to prove this. Furthermore, elevated LH stimulates the luteal gland to produce progesterone and estrogen. Elevated estrogen is directly related to hepatic steatosis and insulin resistance (77). In addition, women with PCOS have androgen excess, IR, variable amounts of estrogen exposure, and many environmental factors, all of which can affect lipid metabolism (78) (79) (76). So further studies are needed to uncover the relationship between LH and lipid metabolism.

Nevertheless, we can infer that there may be a positive association between LH and MAFLD. LH may be related to metabolic diseases by playing an important role in energy metabolism. However, there is currently limited research on the relationship between LH and MAFLD. The specific relationship between LH and MAFLD should be further investigated in future studies.

### PRL

Prolactin (PRL) is an important multifunctional pituitary hormone and involved in several biological functions (80), most notably the promotion of lactation (81). PRL can also stimulate β-cell proliferation and improve insulin secretion, as well as participating in the regulation of glucose metabolism (82, 83). Furthermore, PRL activates the peroxisome proliferator-activated receptor γ (PPARγ) to inhibit lipolysis and activate adipocyte differentiation. Wang et al. found that PRL levels are associated with the lipid metabolism and low-grade inflammatory markers in obesity (84). Studies in PRL receptor-deficient mice have shown increased oxidative stress, SIRT2 expression and apoptosis (85).

Accumulating evidence supports the idea that reduced PRL levels contribute to metabolic changes, and recent studies have found a strong association between PRL and the presence and development of MAFLD (86). Ping Xu et al. found that serum PRL may be a potential biomarker to prevent and treat MAFLD (87). Moderately high PRL levels, both within and above the physiological range, are metabolically beneficial, while extremely high (>100 mg/dL) and extremely low (<7 mg/dL) PRL levels are non-metabolically beneficial (88). In addition, PRL promotes hepatic insulin sensitivity and prevents hepatic steatosis (89–91). The study’s findings suggest that PRL is an important factor in the development of MAFLD.

Recent research has focused on the role of PRL in glucose and lipid metabolism, given its vital role in the maintenance of adipogenesis and adipocyte differentiation. Zhang’s team found that in MAFLD patients, peripheral blood PRL levels are reduced alongside downregulation of hepatic PRL receptor (PRLR) expression (89), while PRL intervention can increase PRLR expression in HepG2 cells. Further studies by Zhang et al. showed that PRL ameliorated hepatic steatosis by inhibiting CD36, suggesting that PRL may protect liver from lipid accumulation via inhibiting CD36 in liver cells, as shown in **Figure 2** (89). Yan et al. reported that prolactin (PRL) acts as a protective factor in MAFLD and that increased PRL levels can improve liver insulin sensitivity. Conversely, decreased PRL secretion can lead to insulin resistance (92). Mechanistically, PRL targets the prolactin receptor (PRLR) and activates the downstream signal transducer and activator of transcription-5 (STAT5) to mediate insulin sensitization (93). Furthermore, PRLR expression is regulated by liver insulin resistance/sensitivity levels, and it is down-regulated in the insulin-resistant state and up-regulated in the insulin-sensitive state (90).

Overall, it can be concluded that PRL has a protective effect on the liver by improving its insulin sensitivity. It also prevents the fat accumulation in the liver. These findings have important implications for the potential use of PRL as a therapeutic target MAFLD.

### ACTH

ACTH is also synthesized by the anterior pituitary gland. The effects of ACTH are mediated by ACTH receptors (ACTHRs), such as MC2R and MC5R.The expression of MC2R and MC5R proteins in mouse embryos was examined (94), and ACTHR mRNA was found to be present in various tissues, including mouse adipose tissue (95), skin (96, 97), mouse pituitary glands (98), rat sympathetic ganglia (99), mouse fetus and new-born testis (100) and human uterine endometrium (100). Besides ACTHR mRNA has been detected in human erythroblasts (101) and human bone marrow cells (102).

Relatively few articles directly describe the relationship between ACTH and MAFLD. It has been reported that three months of exposure to noise at 75 dB SPL was sufficient to exacerbate the progression of MAFLD in mice, with the activation of the hypothalamic–pituitary–adrenal (HPA) axis playing a critical role (103). In depression and CDAHFD-fed mice, hepatic steatosis was aggravated by activating the HPA axis (104) In clinical practice, in patients with insulin-dependent hyperglycemia and concomitant hyperprolactinemia, liver steatosis was reversed and triglyceride levels returned to normal or near-normal levels after treatment with the ACTH receptor antagonist mifepristone (105). Another clinical study also found that in male patients with idiopathic hypogonadotropic hypogonadism (IHH), there is an independent association between MAFLD and ACTH levels (106).

Basic experiments have shown that ACTH significantly inhibits high-density lipoprotein but has no effect on low-density lipoprotein. This suggests that ACTH primarily reduces intracellular lipoproteins (107, 108). ACTH plays an important role in controlling adrenal steroidogenesis. It also induces the expression of mitochondrial superoxide dismutase 2 (SOD2), which plays a role in the removal of ROS from the mitochondria (109). Thus, ACTH modulates the expression of enzymes involved in the biosynthesis of steroids and non-steroids, helping to prevent ROS-induced cell toxicity (110).

Under the stimulation of ACTH, free cholesterol is released from hydrolyzed lipid droplets, increasing steroid production. ACTH stimulates lipolysis, and ACTH treatment has a significant effect on lowering cholesterol levels (111). Current evidence suggests that the cholesterol-lowering effect of adrenal corticosteroid stimulation may be mediated by promoting the hepatic uptake of apolipoprotein B (ApoB)-rich lipoproteins. In HepG2 cells, Xu et al. found that ACTH could reduce the concentration of ApoB-containing lipoproteins in human plasma, suggesting that the main mechanism by which ACTH reduces cholesterol levels *in vivo* may be by reducing the rate of production of ApoB lipoproteins in the liver (112).

Thereby we can conclude that ACTH may play a role in MAFLD by regulating the lipoproteins.

### GH

Growth hormone (GH) is essential for growth and development. It regulates growth, tissue remodeling, extracellular matrix formation and fibrosis. As a key regulator of body composition, GH plays a vital role in maintaining metabolic homeostasis in several organs, including the liver, skeletal muscle and adipose tissue. During fasting and stress, GH plays a key role in anabolic processes. GH acts in both direct and indirect ways: it binds to the growth hormone receptor (GHR) to active downstream signaling directly, it stimulates the expression of insulin-like growth factors (IGFs) and their binding proteins (IGFBP) indirectly to mediate metabolism (113, 114). Metabolically, GH can stimulate lipolysis in white adipose tissue and impair hepatic and peripheral insulin sensitivity (113).

The relationship between MAFLD and GH was investigated. Increased risk of MAFLD/NASH associated with growth hormone axis abnormalities (115). In healthy Asian individuals, individuals with low GH levels had a higher prevalence of MAFLD, there may be a negative correlation between GH levels and MAFLD (116). In line with this is MAFLD usually develops soon after the diagnosis of adult growth hormone deficiency (AGHD). It can progress to non-alcoholic steatohepatitis (NASH) with advanced fibrosis rapidly, and eventually require liver transplantation (117). Growth hormone deficient rats also have MAFLD (118). GH has been used as a drug in the clinic. Clinical studies have shown that administration of higher doses of GH can help overcome the GH resistance in cirrhotic patients and significantly increase serum IGF-I levels (119). Excess GH can reduce total fat mass and hepatic lipid content and induce insulin resistance.

GH can affect the liver by making it insulin resistant. Almost 90 years ago, the Argentinian clinical physician Bernardo Houssay discovered that GH can inhibit insulin action (120). In adipose tissue, GH can inhibit insulin action, thereby reducing glucose uptake, and promoting hepatic gluconeogenesis. Subsequent research has shown that GH has direct and indirect effects on glucose metabolism, with the indirect effects primarily being mediated by IGF-1 (121, 122). GH can also activate the JAK2-STAT2 signaling pathway by targeting the GH receptor, thereby stimulating the synthesis and secretion of insulin-like growth factors (123). Furthermore, GH is involved in pathways associated with late-stage fibrosis in MAFLD, including the TGF-β and MAPK pathways (124, 125).

The most prominent metabolic effects of GH are increased lipolysis and FFA levels. Further studies of longer duration are needed to confirm the potential effects of GH on MAFLD.

### MSH

Melanocyte-stimulating hormone (MSH), which is derived from pro-opiomelanocortin (POMC), plays an important role in the regulation of metabolic functions (126). MSH can be divided into three subtypes: α-MSH, β-MSH, and γ-MSH (127). MSH binds to receptors on the surface cells, to stimulate melanocyte formation and activity of melanocytes, thereby promoting melanin synthesis and affecting the color of the skin, hair, and eye color. α-MSH is a peptide that can suppress people’s appetite and reduce food intake (128). However, in some obese individuals, lower levels of α-MSH expression may cause a disorder in energy balance, this may cause a disorder in energy balance (129), suggesting a potential link between α-MSH and obesity.

MSH also has various functions, most important, Melanocortin peptides have long been thought to be potent inhibitors of inflammation. They are a promising source of new anti-inflammatory and cytoprotective therapies (130). MSH’s effect on MAFLD may be due to its ability to alleviate oxidative stress and exert potent anti-inflammatory effects. α-MSH prevents liver inflammation and injury induced by LPS and paracetamol (131, 132). Furthermore, both *in vitro* and *in vivo* studies have confirmed that MSH reduces pro-inflammatory mediator levels by inducing cyclic adenosine monophosphate (cAMP) and inhibiting nuclear factors (133). Due to the antioxidant and anti-inflammatory characteristics of MSH, it can be used to promote melanin synthesis in adipose tissue. This reduces the generation of ROS and inflammation and prevents the sequelae of obesity. This is beneficial in the prevention of MAFLD.

There is increasing evidence that MSH may also have anti-fibrotic properties. For example, α-MSH has been shown to reduce endotoxin-induced liver inflammation (131), and in α-MSH gene-treated rats, hepatic stellate cells (HSC) and Kupffer cells were significantly inhibited (134). A recent report suggests that α-MSH can regulate collagen metabolism (135). Furthermore, α-MSH has been shown to increase collagen degradation by activating matrix metalloproteinase-1 (MMP-1) and MMP-2 (136, 137). Lee et al. recently found that MSH gene therapy can reverse liver fibrosis in mice treated with carbon tetrachloride for 10 weeks (138). In addition, MSH gene therapy increases the expression and/or activity of MMP-1, MMP -2 and MMP -8.

Furthermore, MSH treatment differentially regulates genes involved in lipid and carbohydrate metabolism in liver and adipose tissue based on their synthesis/degradation metabolic functions (139). Taken together, it can be concluded that MSH may have a beneficial impact on MAFLD through multiple effects, including alleviating oxidative stress and inflammation, scavenging reactive oxygen species, and regulating nutrient metabolism. These findings also provide a new therapeutic approach for treating human fibrotic diseases with MSH and related peptides.

## Conclusion and future perspectives

MAFLD is a multifactorial disease characterized by excessive fat accumulation in the liver. The pathogenesis of MAFLD is complex and closely linked to glucose and lipid metabolism in the liver. In addition to disturbances in glucose and lipid metabolism, pituitary dysfunction is an important mechanism underlying the development of MAFLD. Recent studies have shown that anterior pituitary hormones may regulate hepatocytes independently of target gland hormones. These hormones may influence the development of MAFLD through various mechanisms. Besides, some current clinical therapeutic strategies for MAFLD can affect pituitary hormones. Glucagon-like peptide-1 receptor agonists (GLP-1 RAs) are renowned for their effectiveness in controlling blood sugar levels and managing weight. But the literature suggests that GLP-1Rs may modulate thyroid hormone production and secretion. For example, Ye et al. demonstrated that treatment with liraglutide reduced TSH levels and improved hepatic thyroid hormone resistance in a population of 49 diabetic patients with MAFLD (140). GLP-1 RAs may exert a direct inhibitory effect on the central nervous system, since GLP-1Rs are present in the paraventricular nucleus (PVN) of the hypothalamus, which contains thyrotropin-releasing hormone (TRH)-producing neurons. This suggests that GLP-1 RAs may influence TRH-producing neuron activity directly in the hypothalamus (141). Though, the causal relationship and underlying mechanisms between anterior pituitary dysfunction and MAFLD are still under investigation and require additional data from clinical and basic research. In the future, the impact of MAFLD clinical treatment on pituitary hormone secretion should also be considered, as this could have implications for the body’s endocrine system.
